# Supplementary material for: Age and loneliness relate to reduced trust learning and alterations in amygdala function
Source: iScience. 2026 Jul 11;29(8):116747. doi: 10.1016/j.isci.2026.116747 (PMC13382064; doi:10.1016/j.isci.2026.116747)
Supplement: Document S1. Figures S1 and S2 and Table S1 [file mmc1.pdf]

iScience, Volume *29*

Supplemental information

Age and loneliness relate to reduced trust learning and alterations in amygdala function

Ronald Sladky, Federica Riva, and Claus Lamm

Supplementary Results

Functional MRI

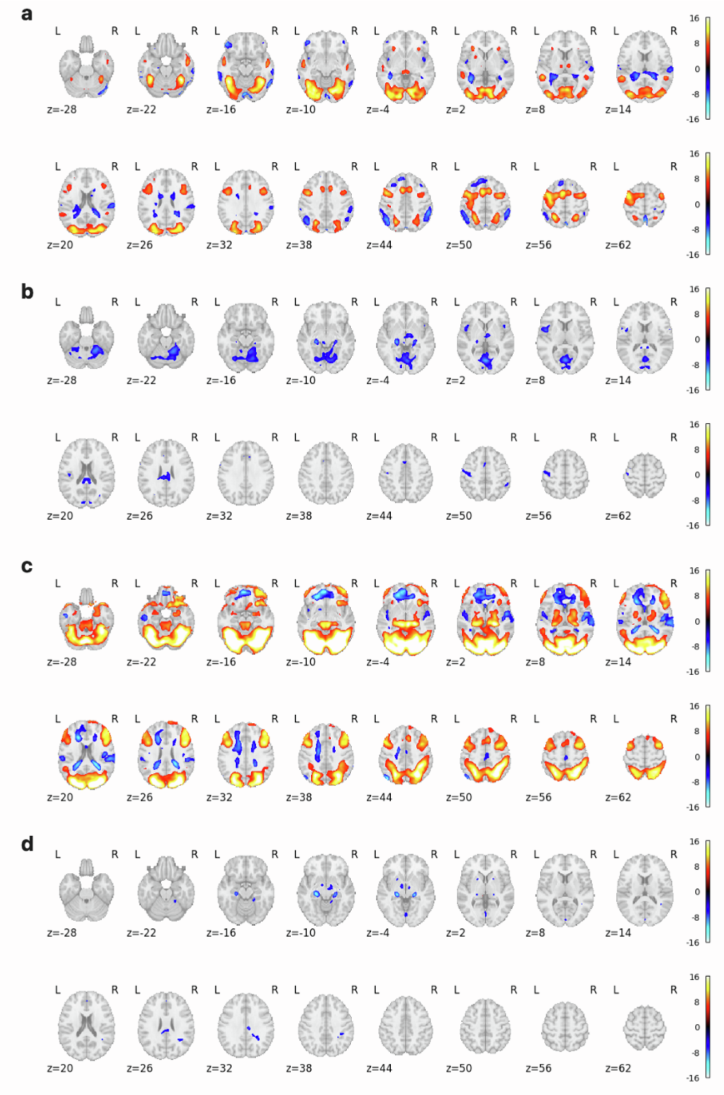

Figure S1: Whole brain fMRI results. **a.** Preparation phase. Activaton in visual areas and thalamus, temporal lobe, anterior insula, and (dorsolateral) prefrontal cortex are consistent with demanding stochastic visual-cognitive decision making. **b.** Age effects during preparation phase. In addition to the hypothesis-driven findings, we observed age-related lower activity in the interoception-related anterior insula and memory-related posterior hippocampus, which could contribute to the lower efficiency of trust behavior in older adults. **c.** Outcome phase. Activation was comparable to the preparation phase but stronger, particularly in the amygdala region, posterior hippocampus, midbrain, and dorsal striatum consistent with reward processing. Dorsomedial prefrontal cortex was hypOctive. **d.** Age effects during outcome phase. Most prominent age-related lower activity were observed in the posterior hippocampus. All $p<0.05$ FWE whole brain corrected. Loneliness or Age*Loneliness interaction were not significant using strict thresholding.

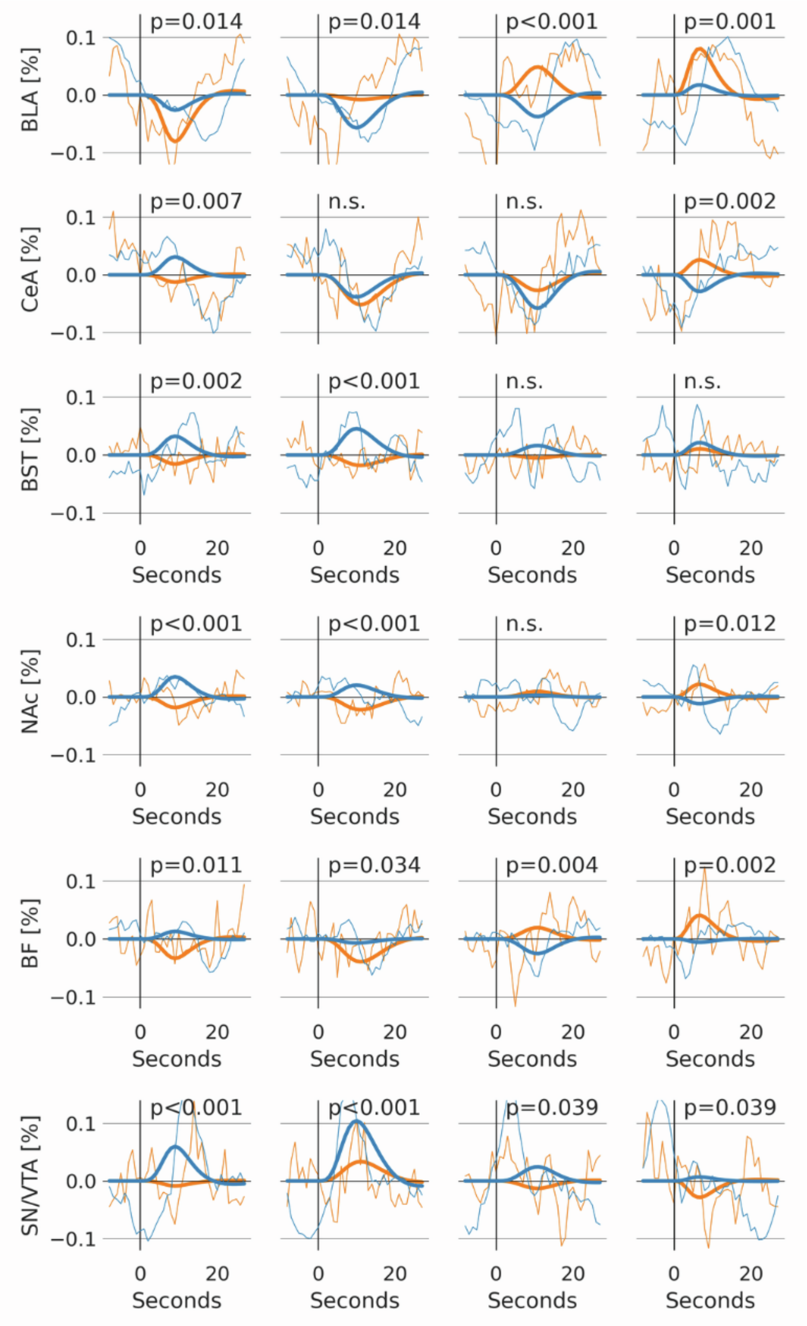

Figure S2: Regional BOLD response in the Volumes of Interest during Preparation, Investment, Waiting, and Outcome phases. Thick lines represent the canonical hemodynamic response model, thin lines the mean BOLD signal. Orange: Older Adults, Blue: Young Adults.

HGF Model

	~ age.group * loneliness.group	~ age.group * loneliness.group * Education	~ age.group * UCLA_Loneliness
μ_0	age.group, F(1,110) = 3082.27, p < .001, η^2(partial) = 0.97<br>loneliness.group, F(1, 110) = 66.90, p < .001, η^2(partial) = 0.38<br>age.group and loneliness.group, F(1, 110) = 1.26, p = 0.264, η^2(partial) = 0.01	age.group, F(1,106) = 3024.39, p < .001, η^2(partial) = 0.97<br>loneliness.group, F(1, 106) = 65.65, p < .001, η^2(partial) = 0.38<br>age.group and loneliness.group, F(1, 106) = 1.24, p = 0.269, η^2(partial) = 0.01<br>Education, F(1, 106) = 0.70, p = 0.404, η^2(partial) = 6.57e-03<br>age.group and Education, F(1, 106) = 0.18, p = 0.674, η^2(partial) = 1.68e-03<br>loneliness.group and Education, F(1, 106) = 0.64, p = 0.426, η^2(partial) = 6.00e-03<br>age.group, loneliness.group and Education, F(1, 106) = 0.42, p = 0.520, η^2(partial) = 3.92e-03	age.group, F(1, 110) = 2665.76, p < .001, η^2(partial) = 0.96)<br>UCLA_Loneliness, F(1, 110) = 41.23, p < .001, η^2(partial) = 0.27)<br>age.group and UCLA_Loneliness, F(1, 110) = 2.86, p = 0.094, η^2(partial) = 0.03
ψ	age.group, F(1, 110) = 32.87, p < .001, η^2(partial) = 0.23<br>loneliness.group, F(1, 110) = 4.34, p = 0.040, η^2(partial) = 0.04<br>age.group and loneliness.group, F(1, 110) = 11.72, p < .001, η^2(partial) = 0.10	age.group, F(1, 106) = 32.31, p < .001, η^2(partial) = 0.23<br>loneliness.group, F(1, 106) = 4.27, p = 0.041, η^2(partial) = 0.04<br>age.group and loneliness.group, F(1, 106) = 11.52, p < .001, η^2(partial) = 0.10<br>Education, F(1, 106) = 0.31, p = 0.579, η^2(partial) = 2.92e-03)<br>age.group and Education, F(1, 106) = 1.08, p = 0.301, η^2(partial) = 0.01)<br>loneliness.group and Education, F(1, 106) = 0.59, p = 0.444, η^2(partial) = 5.55e-03)<br>age.group, loneliness.group and Education, F(1, 106) = 0.14, p = 0.705, η^2(partial) = 1.36e-03)	age.group, F(1, 110) = 32.01, p < .001, η^2(partial) = 0.23<br>UCLA_Loneliness, F(1, 110) = 6.21, p = 0.014, η^2(partial) = 0.05<br>age.group and UCLA_Loneliness, F(1, 110) = 6.53, p = 0.012, η^2(partial) = 0.06
ω_{mean}	age.group, F(1, 110) = 41.84, p < .001, η^2(partial) = 0.28<br>loneliness.group, F(1, 110) = 29.43, p < .001, η^2(partial) = 0.21<br>age.group and loneliness.group, F(1, 110) = 25.17, p < .001, η^2(partial) = 0.19	age.group, F(1, 106) = 41.97, p < .001, η^2(partial) = 0.28<br>loneliness.group, F(1, 106) = 29.53, p < .001, η^2(partial) = 0.22<br>age.group and loneliness.group, F(1, 106) = 25.27, p < .001, η^2(partial) = 0.19<br>Education, F(1, 106) = 3.07, p = 0.083, η^2(partial) = 0.03)<br>age.group and Education, F(1, 106) = 0.40, p = 0.529, η^2(partial) = 3.75e-03)<br>loneliness.group and Education, F(1, 106) = 0.63, p = 0.431, η^2(partial) = 5.87e-03)<br>age.group, loneliness.group and Education, F(1, 106) = 0.24, p = 0.628, η^2(partial) = 2.22e-03)	age.group, F(1, 110) = 31.61, p < .001, η^2(partial) = 0.22<br>UCLA_Loneliness, F(1, 110) = 6.22, p = 0.014, η^2(partial) = 0.05<br>age.group and UCLA_Loneliness, F(1, 110) = 8.12, p = 0.005, η^2(partial) = 0.07
ω_1	age.group, F(1, 110) = 86.53, p < .001, η^2(partial) = 0.44<br>loneliness.group, F(1, 110) = 131.11, p < .001, η^2(partial) = 0.54<br>age.group and loneliness.group, F(1, 110) = 62.83, p < .001, η^2(partial) = 0.36	age.group, F(1, 106) = 87.18, p < .001, η^2(partial) = 0.45<br>loneliness.group, F(1, 106) = 132.09, p < .001, η^2(partial) = 0.55<br>age.group and loneliness.group, F(1, 106) = 63.33, p < .001, η^2(partial) = 0.37<br>age.group and Education, F(1, 106) = 0.55, p = 0.462, η^2(partial) = 5.12e-03<br>loneliness.group and Education, F(1, 106) = 0.64, p = 0.426, η^2(partial) = 5.98e-03<br>age.group, loneliness.group and Education, F(1, 106) = 0.23, p = 0.634, η^2(partial) = 2.15e-03	age.group, F(1, 110) = 44.00, p < .001, η^2(partial) = 0.29<br>UCLA_Loneliness, F(1, 110) = 28.57, p < .001, η^2(partial) = 0.21<br>age.group and UCLA_Loneliness, F(1, 110) = 15.97, p < .001, η^2(partial) = 0.13
ω_2	age.group, F(1, 110) = 19.84, p < .001, η^2(partial) = 0.15<br>loneliness.group, F(1, 110) = 1.53, p = 0.219, η^2(partial) = 0.01<br>age.group and loneliness.group, F(1, 110) = 8.80, p = 0.004, η^2(partial) = 0.07	age.group, F(1, 106) = 19.83, p < .001, η^2(partial) = 0.16<br>loneliness.group, F(1, 106) = 1.53, p = 0.219, η^2(partial) = 0.01<br>age.group and loneliness.group, F(1, 106) = 8.81, p = 0.004, η^2(partial) = 0.08<br>Education, F(1, 106) = 2.79, p = 0.098, η^2(partial) = 0.03<br>age.group and Education, F(1, 106) = 0.30, p = 0.583, η^2(partial) = 2.86e-03<br>loneliness.group and Education, F(1, 106) = 0.60, p = 0.439, η^2(partial) = 5.67e-03<br>age.group, loneliness.group and Education, F(1, 106) = 0.24, p = 0.628, η^2(partial) = 2.23e-03	age.group, F(1, 110) = 18.57, p < .001, η^2(partial) = 0.14<br>UCLA_Loneliness, F(1, 110) = 0.10, p = 0.748, η^2(partial) = 9.41e-04<br>age.group and CLA_Loneliness, F(1, 110) = 2.55, p = 0.113, η^2(partial) = 0.02

Table S1
